# Tumeur de Krukenberg: à propos de 5 cas

**DOI:** 10.11604/pamj.2019.34.106.18928

**Published:** 2019-10-23

**Authors:** Khaoula El Montacer, Fouad Haddad, Fz Kharbachi, Mohammed Tahiri, Wafaa Hliwa, Ahmed Bellabah, Wafaa Badre

**Affiliations:** 1 Service d'Hépato-Gastro-Entérologie, Ibn Rochd, Casablanca, Maroc

**Keywords:** Tumeur de Krukenberg, néoplasie digestive, pronostic, Krukenberg tumor, digestive tract cancer, prognosis

## Abstract

Les tumeurs de Krukenberg sont des tumeurs malignes rares de l'ovaire, souvent bilatérales et secondaires à un cancer volontiers gastrique, muco-sécrétant dans 90% des cas. Nous avions colligé sur une période de 17ans, entre janvier 2002 et janvier 2019, 5 cas de tumeurs de Krukenberg pris en charge dans le Service d'Hépato-Gastro-Entérologie d'IBN ROCHD de Casablanca. L'objectif de cette étude rétrospective et descriptive était de mettre le point sur ce type de néoplasie au pronostic sombre afin d'améliorer les performances diagnostiques et thérapeutiques. L'âge moyen de nos patientes était de 42ans avec des extrêmes entre 25ans et 61ans. L'ascite était la présentation dominante, rapportée dans 80% des cas. Le bilan radiologique avait mis en évidence la tumeur ovarienne de taille d'écho-structure variables, qui était unilatérale dans 60% des cas. La fibroscopie oeso-gastroduodénale avait retrouvé un processus gastrique chez 4 patientes. L'exploration chirurgicale et l'étude immuno-histochimique des prélèvements biopsiques avaient redressé le diagnostic vers un cancer du colon transverse avec une extension locorégionale massive et infiltration gastrique dans un des 4 cas. Et la laparotomie exploratrice du cinquième cas avait retrouvé une néoplasie colique métastatique. La chirurgie radicale n'était possible que chez 2 patientes, dans les 2 autres cas, l'état local avancé n'avait permis que la réalisation des biopsies. Et chez une patiente, l'état général profondément altéré et le haut risque anesthésique ont rendu tout geste chirurgical inutile. Uniquement deux patientes avaient reçu une chimiothérapie postopératoire. L'évolution était fatale dans 100% des cas. Ce travail reconfirme le pronostic catastrophique de la tumeur de Krukenberg vue son évolution souvent insidieuse amenant à un diagnostic tardif et l'incompréhension claire de son étiopathogénie. Ainsi déduisons-nous que l'amélioration des chances de survie repose sur l'exploration systématique des ovaires devant toute néoplasie digestive. Certains auteurs proposent même une ovariectomie prophylactique chez les femmes âgées de plus de 40 ans qui ont été opérées pour une tumeur digestive.

## Abstract (en)

Krukenberg tumours are rare, often bilateral ovarian malignant tumours secondary to muco-secreting gastric cancer, in 90% of cases. We collected data from patients' medical records over a period of 17years, between January 2002 and January 2019. These patients had Krukenberg tumors for which they were treated in the Department of Hepato-Gastro-Enterology at the Ibn Rochd Hospital University. The purpose of this retrospective, descriptive study was to update the current understanding of this type of neoplasm characterized by poor prognosis, in order to improve diagnostic performance and therapeutic treatment. The average age of our patients was 42 years, ranging between 25 and 61 years. Ascites was the most common manifestation and was reported in 80% of cases. Radiological assessment highlighted ovarian tumor of variable size and echo structure, unilateral in 60% of cases. Oeso-gastroduodenal fibroscopy showed gastric process in 4 patients. Surgical exploration and immunohistochemical examination of biopsic specimens resulted in the correct diagnosis of cancer of the transverse colon with massive locoregional extension and gastric infiltration in one case. The fifth patient underwent exploratory laparotomy which revealed metastatic colonic neoplasia. Radical surgery could be performed only in two patients, in the other two cases only biopsies were performed due to the late stage of the disease. One patient had profoundly altered general state and was at high-risk for anaesthetic, then surgical procedure could not be performed. Only two patients received postoperative chemotherapy. Fatal outcome was reported in 100% of cases. This study reconfirms the catastrophic prognosis of Krukenberg tumor based on its insidious evolution often leading to late diagnosis and to a clear misunderstanding of its etiopathogenesis. We conclude that the improvement of survival chances is based on systematic assessment of ovaries in patients with digestive neoplasia. Some authors also affirm that prophylactic ovariectomy should be performed in women older than 40 years who have undergone gastrointestinal cancer surgery.

## Introduction

Les tumeurs de Krukenberg sont une entité spécifique de métastases ovariennes. Le primitif est dans 90% des cas d'origine digestive. Elles sont très rares à étiopathogénie toujours mal élucidée [1]. Nous rapportons une série de 5 cas de tumeurs de Krukenberg répertoriées au Service d'Hépato-gastro-entérologie du Centre Hospitalier Universitaire (CHU) Ibn Rochd de Casablanca. A travers ces observations, nous essayerons de tracer les caractéristiques de cette tumeur mystérieuse et particulière dans ses aspects épidémiologiques, diagnostiques, thérapeutiques et pronostiques.

## Méthodes

Nous avons colligé sur une période de 17 ans, entre janvier 2002 et janvier 2019, 5 observations de tumeurs de Krukenberg secondaires à une néoplasie digestive qui ont été suivies au Service d'Hépato-Gastro-Entérologie du CHU Ibn Rochd de Casablanca. Bien qu'elle soit décrite il y a plus d'un siècle, en 1895 par Friedrich Krukenberg et que plusieurs revues aient été publiées sur le sujet, l'étiopathogénie de cette tumeur métastatique reste toujours mal élucidée. L'objectif de cet article est de sensibiliser les praticiens aux difficultés de prise en charge diagnostique et thérapeutique de cette maladie afin d'améliorer son pronostic sombre.

**Cas 1:** madame FH, âgée de 37ans, ne présentant pas de comorbidités, n'ayant aucune notion de contage tuberculeux récent dans l'entourage, n'était jamais traitée pour une tuberculose maladie et n'avait aucun antécédent de néoplasie personnelle ni familiale. Elle était hospitalisée dans notre formation pour prise en charge diagnostique d'une distension abdominale gênant la respiration associée à des épigastralgies intenses chroniques non irradiantes évoluant depuis 2 mois avant son séjour hospitalier avec une altération de l'état général. L'examen clinique avait retrouvé une patiente ayant un *performance status* (PS) à 2, apyrétique avec une ascite de grande abondance empêchant la palpation de toute masse abdominale profonde ou d'organomégalie. L'échographie abdominale complétée d'une tomodensitométrie (TDM) abdomino-pelvienne avaient objectivé une masse pelvienne de 15cm, des nodules péritonéaux et une ascite de grande abondance. La fibroscopie oeso-gastroduodénale (FOGD) était sans particularités. L'antigène carcino-embryonnaire (ACE) était élevé à 283 UI/ml. La malade avait bénéficié d'une hystérectomie subtotale sans conservation des annexes et une omentectomie. L'étude anatomo-pathologique de la pièce opératoire était en faveur d'une localisation ovarienne d'un adénocarcinome mucineux d'origine colique. La patiente est décédée à 3 mois du début d'une chimiothérapie palliative postopératoire.

**Cas 2:** madame KM, âgée de 61ans, sans antécédents pathologiques notables ni infectieux ni néoplasiques, hospitalisée pour une distension abdominale associée à des douleurs pelviennes, des épigastralgies atypiques et des vomissements, évoluant depuis 10 mois avec une altération de l'état général. L'examen clinique abdominal était gêné par l'ascite de grande abondance chez une patiente ayant un PS à 3, sans hépatomégalie ni splénomégalie ni adénopathies périphériques. La FOGD avait objectivé un processus ulcéro-bourgeonnant antral. La TDM thoraco-abdomino-pelvienne avait rapporté une tumeur ovarienne bilatérale solido-kystique avec une ascite d'allure gélatineuse. Le CA125 était élevé à 147,3 UI/ml. La laparotomie avait retrouvé deux masses ovariennes solides, un processus tumoral au niveau du colon transverse avec un envahissement locorégional et une ascite de moyenne abondance. Nous avions procédé à une hystérectomie totale sans conservation des annexes et l'examen anatomo-pathologique de la pièce opératoire avait posé le diagnostic d'une localisation ovarienne d'un adénocarcinome moyennement différencié d'origine colique. La patiente était considérée à ce stade en dehors de toute ressource thérapeutique et était décédée 1 mois après que le diagnostic ait été posé.

**Cas 3:** madame TM, âgée de 46 ans n'ayant aucun antécédent carcinologique personnel ni familial, qui s'était présentée pour une distension abdominale associée à des douleurs pelviennes et épigastriques atypiques ainsi qu'un pyrosis évoluant 7 mois avant sa consultation avec une altération de l'état général. La patiente avait à son admission un PS à 3 et une ascite gênant l'examen clinique. La FOGD avait mis en évidence un bourgeon de 3cm au niveau de la grande courbure. Les examens radiologiques (échographie et TDM abdomino-pelvienne) avaient retrouvé une tumeur ovarienne droite solido-kystique avec un envahissement utérin, rectal et vésical, un épaississement de 14mm localisé au niveau de la région antrale gastrique sans extension locorégionale et une ascite de moyenne abondance. Le CA125 était élevé à 334,82UI/ml. La patiente avait bénéficié d'une laparotomie exploratrice et l'état local avancé n'avait permis que des biopsies multiples dont l'examen anatomo-pathologique était en faveur d'une localisation ovarienne d'un adénocarcinome moyennement différencié d'origine gastrique. La patiente n'était candidate à aucun protocole thérapeutique et l'évolution était fatale à 2 mois de son geste chirurgical.

**Cas 4:** madame ZF, âgée de 41 ans, se plaignant de douleurs pelviennes associées à des épigastralgies atypiques et des vomissements évoluant depuis 10 mois de son admission au service dans un contexte d'altération de l'état général. L'examen clinique avait retrouvé une malade asthénique avec un PS à 3, une matité des flancs. Aucune masse n'était perçue à la palpation abdominale. La FOGD avait objectivé un ulcère creusant de la petite courbure à bords oedématiés, durs à la biopsie. La TDM thoraco-abdominopelvienne avait retrouvé une tumeur ovarienne bilatérale de 6cm associée à une urétéro-hydronéphrose droite, un nodule hépatique du segment VI d'allure secondaire et une ascite de grande abondance. Le CA125 était augmenté à 412,3UI/ml. A la laparotomie, des masses pelviennes bilatérales au dépend de l'ovaire solides bourgeonnantes blanchâtres et dures de 6cm de grand axe chacune, envahissant l'utérus, la vessie et le rectum associées à des granulations péritonéales. L'analyse histologique complétée par une étude immuno-histochimique avait révélé une localisation ovarienne bilatérale d'un adénocarcinome moyennement différencié d'origine gastrique. La patiente était décédée après sa première cure de chimiothérapie.

**Cas 5:** madame BH, âgée de 25 ans sans historique médical particulier, hospitalisée pour prise en charge de vomissements alimentaires initialement, devenus bilieux associés à des épigastralgies intenses. Le tableau a évolué vers l'installation d'une dysphagie basse aux solides et des méléna, ceci évoluait depuis 40 jours avant son admission, avec une altération de l'état général. L'examen clinique avait retrouvé une malade asthénique, ayant une sensibilité épigastrique et un ganglion de troisier ferme d'environ 3cm. La FOGD avait décrit une lésion bourgeonnante ulcéro-hémorragique sous cardiale avec de gros plis fundique. L'examen anatomopathologique montrait un carcinome peu différencié et invasif gastrique. Les marqueurs tumoraux ACE, alpha-foetoprotéine, CA125 et CA 19-9 étaient élevés à, respectivement à 13.4, 260.4, 294.44 et 2792UI/ml. La TDM thoraco-abdominopelvienne avait noté un épanchement pleural gauche de faible abondance, des adénopathies sus-clavières bilatérales, coelio-mésentériques, inter-aortocaves, iliaques primitive droite et externes bilatérales. Il y avait un épaississement pariétal gastrique cardial hémi-circonférentiel et régulier prenant le contraste d'une manière hétérogène à 17,4mm avec une infiltration nodulaire en flammèche diffuse accompagnée d'un épanchement péritonéal de moyenne abondance. Il existait une masse sus-utérine latéralisée à gauche mal limitée hétéro-dense prenant le contraste d'une manière hétérogène en contact intime avec la face antérieure de l'utérus et une perte de liseré graisseux de séparation. L'ensemble du tableau clinico-radiologique et histologique évoquait un cancer de l'estomac classé T4aN3M1 associé à une tumeur de Krukenberg gauche fort probable. L'état profondément altéré de la malade l'avait rendu à haut risque anesthésique. Elle était adressée aux soins palliatifs au service d'oncologie et a été perdue de vue.

## Discussion

La tumeur de Krukenberg est très rare et ne présente que 1 à 2% des tumeurs ovariennes. Elle se définit par les métastases ovariennes uni ou bilatérales d'un épithélium glandulaire, caractérisées par la présence de cellules muco-sécrétantes en «bague à chaton» au niveau ovarien [1]. Décrite pour la première fois en 1895, elle suscite toujours des interrogations; la principale est le mode de dissémination néoplasique qui peut exister entre le cancer primitif et la métastase ovarienne. La propagation est certainement précoce dans de nombreux cas. Les cancers primitifs notamment en ce qui concerne ceux de l'estomac sont en effet de très petite taille alors que la tumeur ovarienne est déjà volumineuse [2]. Cette tumeur touche la femme en période d'activité génitale. La moyenne d'âge rapportée dans la littérature est de 40 ans, ce qui est proche de notre échantillon: l'âge moyen de nos patientes est de 42 ans avec des extrêmes entre 25 ans et 61 ans [3]. Les tumeurs de Krukenberg sont frustres et les signes d'appel spécifiques sont pauvres si bien qu'elles puissent être découvertes en per-opératoire voire même être une surprise à l'examen anatomo-pathologique. Cette découverte est faite dans les 2/3 des cas avant la tumeur primitive, ce qui explique leur mauvais pronostic [3]. Trois de nos patientes se plaignaient certes de douleurs pelviennes mais c'est le tableau digestif qui était dominant et le principal motif de consultation: l'ascite dans 4 cas et la dysphagie associée à une hémorragie digestive haute dans le dernier. Toutes étaient en très mauvais état général à leur admission. La recherche de cellules malignes sur un prélèvement du liquide d'ascite n'était pas effectuée chez nos patientes. Elle permet la mise en évidence, au microscope optique, de cellules en bague à chaton qui secrètent de la mucine, caractéristique de la tumeur de Krukenberg [4]. L'échographie et le scanner objectivent la taille volumineuse de ces tumeurs. Les tumeurs bilatérales sont prépondérantes [3]. L'imagerie montre des masses au dépens de l'ovaire, solido-kystiques multi-lobulaires avec des signes de malignité (l'extension locorégionale, les nodules péritonéaux) mais ne permet en aucun cas de différencier la tumeur ovarienne primitive d'une tumeur secondaire. Elle reste indispensable dans le bilan d'extension [5]. Uniquement deux de nos patientes présentaient une atteinte des deux ovaires. L'étude histologique est la seule à pouvoir affirmer le diagnostic. Microscopiquement, la tumeur de krukenberg se caractérise par la présence de cellules épithéliomateuses en «bague à châton» à noyau excentré remplis de mucus muci-carminophile, isolées ou groupées en amas au sein de fibrilles et par une prolifération pseudo-sarcomateuse du stroma [6]. Dans notre petite série, une patiente seulement n'a pas eu de confirmation histologique du fait de la carcinose péritonéale et l'état général altéré, rendant inutile tout geste chirurgical. Au plan hormonal, le CA 125 est le marqueur le plus fréquemment utilisé par les auteurs dans les tumeurs de Krukenberg. En fait il est le marqueur le plus souvent élevé. Il peut jouer un rôle dans le dépistage précoce des métastases ovariennes, dans le suivi et même le pronostic. Les auteurs ont constaté que la survie est inversement proportionnelle avec le taux du CA125 [4]. L'ensemble de ces constatations sont compatibles et transposables à notre échantillon maigre.

Le cancer primitif est dans 90% des cas d'origine digestive dont 70% gastrique, 14% colique, 5% pancréatico-biliaire et 2% appendiculaire; encore plus rarement mammaire et thyroïdienne [1-7]. Trois de nos patientes présentaient une localisation ovarienne d'un primitif gastrique et les deux autres d'origine colique. Vue la rareté de cette tumeur, les données de la littérature demeurent très limitées à des rapports de cas et ainsi insuffisante pour la bonne formation et information des praticiens. Nos cas rejoignent ceux de Benaaboud *et al*. Seck *et al*. Falandry *et al*. Berthé *et al*. Brahim *et al*. Vauthier-Brouzes *et al.* et Muller *et al.* afin d'enrichir la publication scientifique et améliorer la prise en charge de ces néoplasies redoutables [3], [6], [8-12]. Le traitement est d'abord chirurgical et consiste en une hystérectomie totale sans conservation annexielle (HTSA) avec omentectomie pour la tumeur ovarienne. La tumeur digestive primitive diagnostiquée secondairement serait traitée en fonction de son stade évolutif. Le traitement adjuvant est toujours débattu, certains auteurs proposent l'association contenant les produits tels que Adriamycine, Fluoro-Uracile (5 FU) et Cisplatine, d'autres ont même proposé une immunothérapie. La spécificité de l'hormonothérapie reste encore à établir alors que la radiothérapie est totalement inefficace comme le montre toutes les études publiées actuellement [13]. L'hystérectomie totale sans conservation des annexes était réalisée uniquement chez deux patientes permettant d'établir le diagnostic sur l'analyse anatomopathologique de la pièce opératoire. Les deux autres patientes avaient une extension locorégionale massive de la tumeur la rendant inextirpable ne permettant que la réalisation de biopsies et la dernière présentait un haut risque anesthésique vue l'état général et nutritionnel profondément altérés et n'a même pas été opérée. Uniquement deux patientes ont subi une chimiothérapie postopératoire. A l'heure actuelle et comme le cas pour nos patientes, le pronostic demeure sombre. La moyenne de survie rapportée dans la littérature est de 12 mois après le début du diagnostic [3]. Dans notre série, cette moyenne était réduite à 2 mois. Les facteurs pronostiques les mieux connus sont: le diagnostic tardif, la symptomatologie ovarienne parlante, l'épanchement pleural et/ou péritonéal, la femme jeune en période d'activité génitale, un traitement peu agressif et la mauvaise chronologie des actes chirurgicaux [3]; et toutes nos patientes remplissaient 4 des 6 critères. Ainsi rejoignons-nous nos collègues du Service de Gynécologie Obstétrique B, Maternité Lalla Meryem du CHU Ibn Rochd de Casablanca ayant proposé et publié des recommandations dans le but d'améliorer le taux de survie de ces patientes [3]: l'examen gynécologique systématique chez toute femme atteinte d'un cancer digestif; l'exploration systématique des deux ovaires chez toute femme opérée pour cancer digestif ; la recherche clinique et para clinique systématique, en particulier par une fibroscopie digestive d'un cancer digestif devant une tumeur ovarienne volontiers bilatérale. En outre, Rodier a même présenté en 1992 la place de l'ovariectomie bilatérale prophylactique la préconisant en même temps opératoire de l'exérèse de toute tumeur digestive [14]. Une telle suggestion semble prometteuse dans notre contexte socio-économique pauvre où une surveillance clinico-radiologique des malades ainsi que les modalités de dépistage disponibles peuvent être coûteuses et astreignantes; cependant une proposition de cette ampleur nécessite des évaluations supplémentaires avant de l'adopter dans la prise en charge des tumeurs de Krukenberg.

## Conclusion

Les tumeurs de Krukenberg constituent toujours un véritable défi pour les praticiens; d´où l´intérêt d´un examen gynécologique systématique devant toute néoplasie digestive et réciproquement, une exploration digestive radiologique et endoscopique est jugée également nécessaire devant toute tumeur ovarienne.

### Etat des connaissances actuelles sur le sujet

- Les tumeurs de Krukenberg sont rares et de pronostic sévère;
- L'étiopathogénie demeure mystérieuse limitant ainsi l'amélioration de la prise en charge diagnostique et thérapeutique.

### Contribution de notre étude à la connaissance

- L'exploration chirurgicale est indispensable au diagnostic et pour un éventuel traitement; quand l'état de la malade le permet;
- Des recommandations de dépistage doivent encore être développées afin de remédier au diagnostic tardif;
- L'ovariectomie prophylactique reste un sujet débattu nécessitant plus de réflexion, quoiqu'il semble être à encourager dans notre contexte socio-économique et sanitaire.

## Conflits des intérêts

Les auteurs ne déclarent aucun conflit d’intérêts.
